# Comprehensive Review of Polymer and Polymer Gel Treatments for Natural Gas-Related Conformance Control

**DOI:** 10.3390/gels8060353

**Published:** 2022-06-05

**Authors:** Ali Al Brahim, Baojun Bai, Thomas Schuman

**Affiliations:** 1 Department of Geosciences and Geological and Petroleum Engineering, Missouri University of Science and Technology, 1400 N. Bishop, Rolla, MO 65409, USA; aaagh9@mst.edu (A.A.B.); baib@mst.edu (B.B.); 2 Department of Chemistry, Missouri University of Science and Technology, 400 W. 11th St., Rolla, MO 65409, USA

**Keywords:** polymers, polymer gel systems, natural gas, conformance control, disproportionate permeability reduction

## Abstract

Conformance problems often exist in natural gas-related activities, resulting in excessive water production from natural gas production wells and/or excessive natural gas production from oil production wells. Several mechanical and chemical solutions were reported in the literature to mitigate the conformance problems. Among the chemical solutions, two classes of materials, namely polymer gels and water-soluble polymers, have been mostly reported. These systems have been mainly reviewed in several studies for their applications as water shutoff treatments for oil production wells. Natural gas production wells exhibit different characteristics and have different properties which could impact the performance of the chemical solutions. However, there has not been any work done on reviewing the applications of these systems for the challenging natural gas-related shutoff treatments. This study provides a comprehensive review of the laboratory evaluation and field applications of these systems used for water control in natural gas production wells and gas shutoff in oil production wells, respectively. The first part of the paper reviews the in-situ polymer gel systems, where both organically and inorganically crosslinked systems are discussed. The second part presents the water-soluble polymers with a focus on their disproportionate permeability reduction feature for controlling water in gas production wells. The review paper provides insights into the reservoir conditions, treatment design and intervention, and the success rate of the systems applied. Furthermore, the outcomes of the paper will provide knowledge regarding the limitations of the existing technologies, current challenges, and potential paths forwards.

## 1. Introduction

Excess water and gas production caused by reservoir conformance problems impose detrimental effects on the performance of hydrocarbon production wells. They lead to an increase in the operating and handling costs, sand production, and loss of well productivity. Many mature production wells suffer from conformance problems, such as poor cementing, water coning, gas cusping, and fractures [1,2]. Correcting the conformance problems is crucial for maintaining and extending the productivity of the wells. Many techniques were adopted to treat the conformance problems. These techniques are generally classified as mechanical and chemical solutions.

The mechanical solutions for treating the conformance problems involve the deployment of bridge plugs, packers, and patches [3,4,5]. However, the mechanical solutions require a wellbore with a competent casing. In addition, they are ineffective in treating the conformance problems which are located deeper in the reservoirs [6,7]. The chemical solutions for treating the conformance problems include mainly cement squeeze, epoxy resin, polymers, and polymer gel systems. The cement squeeze and epoxy resin are not preferable because of their poor injectivity, especially for treating matrix-related conformance problems and narrow fractures. In addition, it is challenging to clean the wellbore when the placement technique fails to reach the targeted depth [8,9,10].

Water-soluble polymers and polymer gel systems have several advantages over the other chemical methods, including the ability to tailor the compositions to meet the reservoir conditions, cost-effectiveness, ease of pumping, and penetration into the reservoir [11,12]. The performance of these two systems was broadly highlighted and reviewed for their water shutoff treatments targeting oil production wells [2,13,14,15]. However, natural gas production wells exhibit different characteristics, such as high mobility, low permeability, and high temperature [16]. In addition, the natural gas properties are different, especially in terms of having low viscosity and low density [17]. These parameters significantly affect the performance of these systems. However, there is a lack of review papers that focus on the evaluation and applications of these systems for the challenging natural gas-related shutoff treatments.

**Figure 1:**
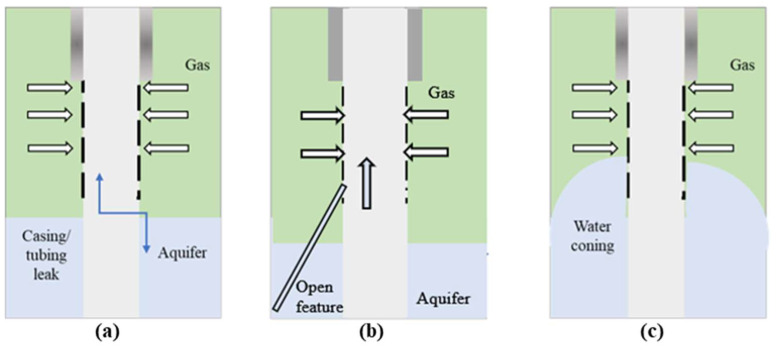
Sources of excess water in gas production wells, (**a**) Casing/tubing leak, (**b**) Open fracture, (**c**) Water coning.

Water shutoff treatments in natural gas production wells (WSOGs) involve the placement of polymer gel systems or polymers to target the source of water influx without sacrificing gas productivity. The water influx invades the natural gas-producing layers through different mechanisms including casing/tubing leak, open feature (fracture or fracture-like channel), and water coning as illustrated in Figure 1. A successful WSOG should tackle the source of the water influx and extend the productivity of the well by significantly reducing the water–gas ratio (WGR) [18,19]. Compared to oil production wells, water shutoff in gas production wells is challenging because as the water invades the gas-producing zone a significant reduction in the available drawdown pressure can occur, leading to a rapid drop in the gas productivity and ultimately the recovery factor [20]. In addition, gas production wells are usually developed in low- to ultralow permeability reservoirs. Thus, there is a higher risk of formation damage during polymer/polymer gel treatments. Once the producing formation is damaged, the gas productivity will be significantly lost. Moreover, polymers and polymer gels have been widely evaluated for their disproportionate permeability reduction (DPR) behavior. However, few studies have been conducted to determine the extent to which polymers and polymer gels can better reduce water permeability than gas permeability. Some of these studies were conducted using nitrogen to replace the natural gas, but such a replacement has not been approved to be applicable.

**Figure 2:**
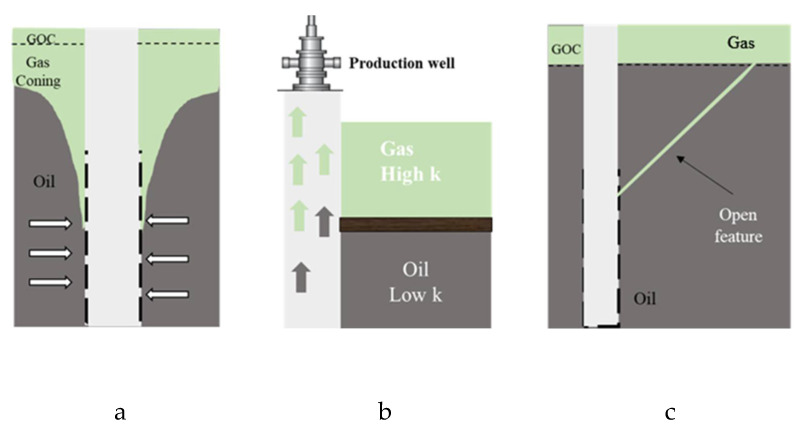
Sources of excess gas in oil production wells, (**a**) Gas coning, (**b**) High permeability contrast, (**c**) Open feature.

Polymer gel systems are also applied as gas shutoff treatments in oil production wells (GSOOs). Potential sources of the unwanted gas production are gas coning in vertical wells, gas channeling that mainly originates from the gas cap [18,21], casing/tubing leak along the wellbore, and excess gas approaching the production well through a high-permeability layer with or without the existence of a barrier to the oil production zone (Figure 2). Controlling the high mobility of natural gas during GSOO is necessary to ensure the well remains under production with an acceptable gas–oil ratio (GOR).

The shutoff treatments are classified as either nonselective or selective. The nonselective treatments involve the placement of a polymer gel system into the target zone, while mechanically or chemically isolating the hydrocarbon-producing intervals. In the selective treatment, the polymer gel systems or polymers are bullheaded (fullbore placement) to the producing intervals with the objective of selectively reducing the permeability of one phase, while preserving the permeability of the other phase, and that depends on the type of fluids withdrawn from the production well.

**Figure 3:**
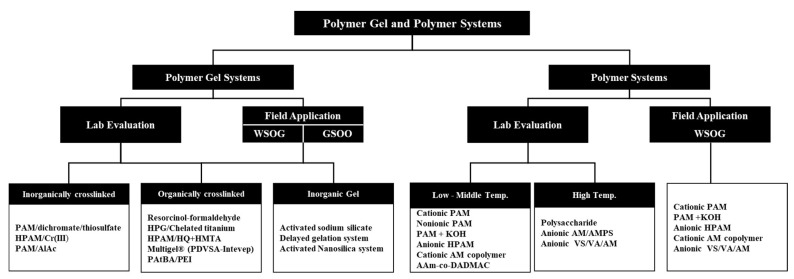
Polymer gel and polymer systems for WSOGs and GSOOs.

This paper provides a comprehensive review of the laboratory evaluation and field applications of using polymer gel systems as WSOGs and GSOOs and polymer systems as WSOGs, as shown in Figure 3. The review of the polymer gel systems summarizes the performance of both inorganically and organically crosslinked systems, as well as the inorganic gel systems. The performance of polymer systems is discussed in the second part of the paper. The polymer systems are classified in this paper as systems studied for low to medium reservoir temperatures and systems evaluated for high-temperature reservoir applications. The paper is concluded with a discussion in which the limitations of the current technologies and evaluation methods are presented. In addition, the discussion provides key points for the future development of systems suitable for natural gas-related shutoff treatments. 

## 2. Polymer Gel Systems

The polymer gel systems mainly consist of two components: polymers and crosslinkers. The gelation of the system is trigged by internal or external stimulation at reservoir conditions, resulting in the formation of a three-dimensional network gel in the target zone [22]. The gelation of these systems occurs inside the target formation; therefore, the rate of gelation is strongly affected by the reservoir conditions such as temperature, pH, and salinity. The systems can be broadly classified based on the crosslinker component as inorganically and organically crosslinked polymer gel systems. The most common inorganically crosslinked system is the hydrolyzed polyacrylamide (HPAM) and chromium acetate (Cr(III)) system, which was patented by Sydansk [23,24]. The formulation of the system can be tailored based on whether the treatment is targeting matrix- or fracture-related conformance problems. The system was claimed to be H_2_S-resistant and has a working temperature of up to 124 °C [25]. Other inorganically crosslinked polymer gel systems reported in the literature include HPAM crosslinked by aluminum citrate [26] and HPAM/zirconium lactate [27].

Organically crosslinked polymer gel systems were reported in the literature for water and gas shutoff treatments in production wells. One of those systems is a copolymer of polyacrylamide tertiary butyl acrylate (PAtBA) crosslinked with polyethyleneimine (PEI) [28]. The system was developed to overcome some issues related to the HPAM/Cr(III) system, such as the rapid gelation at high temperatures and the precipitation of the crosslinker at high pH [29]. The working temperature of the system ranges from 60 to 121 °C. In addition, the system was applied in a lower temperature reservoir (27 to 60 °C) by changing the base polymer of the system and in a higher temperature reservoir (>121 °C) through the incorporation of water-soluble carbonate retarder [30]. However, the incorporation of a retarder into the system could adversely impact the gel strength, and also the retarder could be incompatible with the field mixing water [29]. In addition, the PAtBA/PEI system was claimed to be resistant to acid and stable in H_2_S and CO_2_ environments [31]. Besides the PAtBA/PEI system, other organically crosslinked polymer gel systems applied include hydrolyzed polyacrylamide crosslinked with a combination of hydroquinone and hexamethylenetetramine (HPAM/HQ+HMTA) [32] and the PDVSA-Intevep polymer gel system [33].

The polymer gel systems are usually applied for reservoir formation with high permeability and fractures. However, in order to prevent the risk of damaging the productivity of the treated wells, these polymer gel systems should exhibit low viscosity and high injectivity until reaching the target location. This is particularly true for treating gas production wells, where the formation permeability generally ranges from low to medium. Moreover, the applied polymer gel systems should withstand the high differential pressure located near the wellbore. The cost and the environmental concerns are two other parameters that contribute to the selection of polymer gel systems as shutoff treatments.

### 2.1. Inorganically Crosslinked Polymer Gels

This section provides a review of the polymer gels crosslinked by inorganic crosslinkers such as HPAM/Cr(III) and polyacrylamide (PAM) crosslinked with aluminum acetate (AlAc). The review will be divided into lab evaluation research results and pilot tests, separately. 

#### 2.1.1. Laboratory Evaluation

A limited number of laboratory evaluations were conducted to examine the feasibility of the polymer gel systems for controlling excess water in natural gas production wells, as shown in Table 1. The major parameters tested involve the polymer gel system plugging efficiency, (DPR), which is also referred to as relative permeability modification (RPM) expressed in terms of selectivity; and stability of the polymer gel systems in response to subsequent water and gas cycles (WAG). The plugging efficiency is defined as the maximum pressure drop that the emplaced polymer gel can withstand before water/gas breakthrough occurs. The selectivity is defined as the ratio of the water to gas residual resistance factors (F_rrw_/F_rrg_). A ratio that is significantly greater than 1 implies that a polymer gel system can significantly reduce the water relative permeability (K_rw_) while inducting no or little effect on the gas relative permeability (K_rg_). The stability usually refers to the thermostability under reservoir conditions, which is important for a gel treatment because it is highly related to the effective terms of the treatment. Table 1 summarizes the experimental parameters and the outcomes of the laboratory evaluation of the inorganically crosslinked polymer gel systems.

Dovan and Hutchins [34] screened different types of polymer gels system to determine their selectivity in controlling excess water production for a gas production well with fracture-related conformance problems at 59 °C. The coreflood experiments were conducted using Berea sandstone cores with a permeability (K) ranging from 383 to 541 millidarcy (md). A center tap configuration where the sequential polymer and gas (N_2_) were injected in the middle was employed, and then the effect of the polymer gel on the water and gas permeability reduction was evaluated through linear configuration. The findings of this study indicated that both lignosulfonates/chromium and silicate systems were nonselective. Additionally, both cationic and anionic polyacrylamide gelled systems (4000 ppm) showed a slight selectivity for gas compared to water with F_rrw_/F_rrg_ = 6.7. Seright [35] evaluated the selectivity of 13,900 ppm HPAM/212 ppm Cr(III) gel using a sandstone core with a permeability of 650 md. The polymer gel system was exposed to multiple cycles of water alternating N_2_ gas (WAG). The outcomes of the study showed that F_rrw_ was higher than F_rrg_ for each WAG cycle, but the ratio of F_rrw_/F_rrg_ was progressively decreased due to the polymer gel breakdown. Similar findings were observed in a study conducted by Kantzas et al. [36] to evaluate the applicability of using 5000 ppm HPAM/800 ppm Cr(III) for plugging fractures in tight gas reservoirs. The evaluation was conducted using an artificially fractured carbonate core with a total permeability ranging from 54.3 to 155.9 md. The results showed that the selectivity (F_rrw_/F_rrg_) was 8.75, but the ratio decreased with subsequent cycles due to gel deterioration. Burrafato et al. [37] investigated the selectivity of 35,000 ppm HPAM/900 ppm Cr(III) using a sandstone core with a brine permeability of 170 md. The test showed a reduction in brine permeability by 70% and an increase in the gas permeability. Wawro et al. [19] stated that a similar experiment was conducted to evaluate the selectivity of the HPAM/Cr(III) system using crushed carbonate cores with a permeability range from 10 to 20 darcy. However, the ratio of the system selectivity F_rrw_/F_rrg_ was around 1.5 to 2, which was lower than the results reported by Dovan and Hutchins [34] and Seright [35]. 

Several parameters affecting the HPAM/Cr(III) selectivity performance were examined by Al-Shajalee et al. [39]. Three models were adopted in the study to assess gel/water/gas interactions in the porous media: coreflood, glass micromodel, and capillary tube. The formulation of the gel system applied was 20,000 ppm poly(acrylamide-co-acrylic acid crosslinked with different Cr(III) concentrations (200–600 ppm). The findings of the study showed the selectivity of the gel system was influenced by both water retention and gel lubrication effect mechanisms. Thus, F_rrw_ values showed a shear thinning behavior with respect to injection flowrate, while F_rrg_ values exhibited a shear thickening behavior. In addition, the outcomes of the study implied that the selectivity ratio (F_rrw_/F_rrg_) was reduced with the increase in both the gel rigidly and injection flowrate.

Wassmuth et al. [38] conducted several studies to improve the gel placement during the sequential gel/gas injections. The technique involves the overdisplacement of the gelant from the wellbore with semistable foam, rather than gas. This was studied for resolving the issues encountered in restoring the gas permeability in the natural gas production wells treated with alternate injections of gas (N_2_) and gelant. The performance of this technique was examined using coreflood and sandpack models, and both were treated with HPAM/Cr(III). The results of the study showed that overdisplacing the gelant with foam was efficient resulting in lower F_rrg_ when compared to the gas overdisplacement. However, only some selectivity was observed during the sequential gas and brine after gel treatment.

Shamlooh et al. [40] evaluated the use of different aluminum-based crosslinkers with polyacrylamide systems for the potential use for sour gas conformance control. Four ligands associated with aluminum, namely acetate, amino-acetate, nitrate, and lactate, were tested. The study focused on the performance of these crosslinkers in terms of gelation time and rheological properties under a wide of pH and temperature. The plugging efficiency experiment was carried out using an API permeability plugging tester (PPT), with a fractured disc of 1 mm width. The finding of this study showed that the PAM/AlAc had a better control over the gelation time and sealed the fracture under 700 and 2000 psi.

#### 2.1.2. Field Applications

This section presents a summary of the field applications of using the HPAM/Cr(III) gel as a WSOG (Table 2) and GSOO (Table 3), respectively.

##### HPAM/Cr(III)—Water Shutoff Treatments in Gas Production Wells (WSOGs)

A summary of the reservoir parameters, treatment design, and outcomes of applying HPAM/Cr(III) as water shutoff in gas production wells is presented in Table 2. Alternate injections of gas (N_2_) and gelant were performed to selectively control excess water in fracture-related conformance in gas production wells. The purpose of the N_2_ injection was to generate channels through the injected gelant near the wellbore for natural gas production. Dovan and Hutchins [34] applied the technique in a gas field located in the north of Mexico with a 59 °C bottom hole temperature (BHT). A total of 670 bbl of gelant alternated with three slugs of N_2_ (total = 176 Mscf) were placed with coiled tubing (CT). After the treatment, the well failed to be put back into production due to the resulting polymer damage near the wellbore. After the wellbore was cleaned, the treatment results showed a reduction in water production rate by 93.8%, and the gas production rate was 1900 Mscf/d averaged for three years. The same technique was followed in treating a vertical well with a horizontal sidetrack located in British Columbia, Canada [19]. The objective of the treatment was to control the flow of water influx originating from a fracture located between two formations with direct communication. High-molecular-weight (HMW) HPAM/Cr(III) with a total of 790 bbl of gelant and 625 Mscf nitrogen gas were sequentially bullheaded to the well. The polymer concentrations were staged from 3000 to 8000 ppm. The injection of the high concentration in the last stage served to withstand the high differential pressure of the wellbore. The treatment results indicated that the well failed to produce initially, but the second attempt of putting the well in production showed that water–gas ratio (WGR) decreased by 58%, and there was an increase in the gas production rate by 71% for the period of six months.

HPAM/Cr(III) was also applied for mitigating excess water in a multilayer gas well. [37] reported a field application in treating a multilayer perforated vertical wellbore, which was equipped with an inside casing gravel pack (ICGP). The treatment was designed to occupy the bottom of completion and leave 5 ft at the top of perforation for gas production. The treatment was bullheaded to two perforated intervals with a total thickness of 11 and 30 ft, respectively. A total of 314 bbl of the gel system was injected and overdisplaced with N_2_. The outcomes of treatment showed a reduction in the gas rate by 100% and a decrease in water production rate by 86%. However, after two weeks, the gas rate was reduced to pretreatment level, with a constant WGR. The authors attributed the decline in the gas rate to the low gas reserve presented in the well. 

Brady et al. [41] reported the results of applying HMW HPAM/Cr(III) for reducing water production in a horizontal gas well in the Fayetteville shale. The source of excess water entry was assisted through video production logging. The target zone was mechanically isolated through a composite bridge plug and drillable cement retainer. Then, the treatment was bullheaded to the target formation by sequentially injecting 400 bbl of low concentration polymer (5500 to 8500 ppm at HMW), followed by 40 bbl of a high concentration polymer (50,000 ppm at low molecular weight (LMW)) for tail in. The results of the polymer gel treatment indicated a reduction in the water production rate from 180 to 4 BWPD and a gas production rate improved by 500 Mcf/d.

##### HPAM/Cr(III)—Gas Shutoff Treatments in Oil Production Wells (GSOOs)

Table 3 summarizes the gas shutoff treatments using HPAM/Cr(III). Three wells were treated with high-concentration (40,000 to 50,000 ppm) LMW HPAM/Cr(III) in Prudhoe Bay, Alaska [42]. The objective of the treatment was to squeeze the polymer gel system to shut off gas production from leaking cement-squeezed zones and open perforations located at the top of the oil-producing interval. The BHT of the wells was around 104 °C. Therefore, a precooling of the wellbore was performed to lower the wellbore temperature to 44 °C. The oil-producing interval was protected with CT run with either caped sands or a bridge plug. A total of 100 bbl of the gel system was placed in the target zone with a CT inflatable packer. The results of those treatments indicated a reduction in GOR for the period of six months. Afterward, the GOR ratio returned to the pretreatment level. Based on the diagnostic plots [18], the mechanism of the excess gas was due to a combination of a large gas coning and channeling. In addition, the conducted analysis showed the low success rate of those treatments was due to the low injected volume. It was reported that more than 37 wells were treated with HPAM/Cr(III) gels for gas shutoff treatments in oil production wells located at the Prudhoe Bay Field, AK [42]. Around 60% of those treatments were technically successful for wells with temperatures up to 104 °C. 

A combination of cement and gel squeeze was reported in the literature for treating excess gas production originating from gas channeling to leaking cement-squeezed perforations. The objective of the technique was to provide a robust perforation sealant by using gel for deep matrix (5–10 ft) gas blockage and using cement to fill void spaces. Lai et al. [43] reported the results of combining both HPAM/Cr(III) and cement squeeze for gas shutoff treatment. Thirteen treatments were performed at a candidate reservoir at 8800 ft, 85–99 °C BHT, and gas permeability of 150–300 md. The polymer concentrations for these treatments were 50,000 to 70,000 ppm at a low degree of hydrolysis. Sodium hydroxide (NaOH) was added to the polymer solution to control gelation rate and increase hydrolysis degree. The treatment well intervention involved isolating the oil-producing zone with a sand plug, gelant squeeze with CT, and water injection as a spacer, followed by cement squeeze with CT. The success rate of those treatments was reported to be 86%.

### 2.2. Organically Crosslinked Polymer Gels

#### 2.2.1. Lab Evaluation

Table 4 presents lab studies conducted to evaluate different organically crosslinked polymer gel systems for water shutoff treatments in gas production wells.

Seright [35] evaluated the selectivity of 30,000 ppm resorcinol/30,000 ppm formaldehyde gel using a sandstone core with a permeability of 650 md. The polymer gel system was exposed to multiple cycles of water alternating gas (WAG). The gas injection was 900 psi N_2_. The outcomes of the study showed that the F_rrw_ was higher than F_rrg_ for each WAG cycle, but the ratio of F_rrw_/F_rrg_ significantly decreased due to the polymer gel breakdown.

Several studies were conducted to examine the performance of the PAtBA/PEI gel system. Okasha et al. [44] conducted coreflooding experiments to examine the feasibility of applying the PAtBA/PEI system to treat a deep carbonate reservoir with 90 °C BHT and salinity of 218,000 ppm TDS. The result of the plugging efficiency of the gel system at 2914 md core demonstrated a selectivity of F_rrw_/F_rrg_ = 2.2. The plugging efficiency of PAtBA/PEI was also evaluated using a sandpack model with a permeability of 7.8 md [45]. The sandpack was treated with 1.5 PV of the gel system, and the gel system plugging efficiency to gas was evaluated using N_2_. The gel system endured the gas injection pressure at 865 psi for six hours. Then, the injection pressure dropped and remained stable at 425 psi for 15 hours (F_rrg_ = 6555). In addition, the gel system plugging efficiency was evaluated using a fractured chalk core. The plugging efficiency to seawater showed a breakthrough pressure of 196 psi. Al-Muntasheri et al. [46] conducted a lab study to evaluate a new retarder system to extend the gelation time of the gel system for controlling excess water in a horizontal gas-producing well at 149 °C. The evaluation was carried out to examine the gelation time and the injectivity of the gel system through a tight carbonate core with matrix permeability of 2.75 md. The study showed that the new retarder system was able to delay the gelation of the system by a factor of 3 and improved the injectivity compared to the low concentration system without a retarder system.

#### 2.2.2. Field Applications

Table 5 and Table 6 list the reservoir parameters, treatment design, and outcomes of applying PAtBA/PEI as a WSOG and GSOO, respectively. A summary of other organically crosslinked polymer gel systems which were applied in the field is presented in Table 7.

##### PAtBA/PEI—Water Shutoff Treatments in Gas Production Wells

A summary of the PAtBA/PEI water shutoff treatments is presented in Table 5. 

The PAtBA/PEI system was applied to control excess water production in a multilayer sandstone gas field located in Peciko offshore field, Indonesia (BHT = 150 °C, BHP = 2200) [10]. The treatment was designed for a monobore deviated well with multiple perforated layers. The treatment was injected to 30 ft perforation with a matrix permeability of 500 md. The gas-producing zone was mechanically isolated using a retrievable bridge plug, and the treatment was bullheaded. The gel treatment reduced the water rate from 4000 to 100 BWPD, and the gas rate increased from 2000 to 12,000 Mscf/d. Another monobore vertical well was treated with the gel system in the Gulf of Thailand [47]. A total of 7 bbl of the gel system followed by lost circulation material (LCM) was injected to treat two watered-out perforated zones of 18 ft in thickness. The results of the treatments showed a reduction in the water rate by 49%. The system was also implemented to control water in carbonate formation with a matrix permeability ranging from 2 to 3 md. Al-Muntasheri et al. [46] reported a field application of using the system in controlling water originating from the toe in an open hole horizontal well. The reservoir temperature was 149 °C at 7000 psi, and the total depth was 13,611 ft. The treatment design consisted of the injection of 150 bbl of the gel formulation tailed in with 5 bbl of gel mixed with silica flour. Then, the treatment was shut in for three days. The results of the treatment showed a reduction in water production by 58% and an increase in gas rate from 2200 to 17,000 Mscf/d. However, these reported results were the response of the treated well for the duration of eight days only. 

##### PAtBA/PEI—Gas Shutoff Treatments in Oil Production Wells

Table 6 summarizes the gas shutoff treatments using PAtBA/PEI.

Bach et al. [48] reported a field treatment conducted in the North Sea using PAtBA/PEI system to reduce gas production in a wellbore with an openhole completion, at 88 °C and 3500 psi reservoir conditions. The excess gas was due to a high-permeability streak located between the casing shoe and the top of the gravel pack. The well intervention included running an inflatable plug with CT, followed by injecting 38 bbl of temporary plugging agent to isolate the oil-producing zone. Then, 628 bbl of the gel system was injected through CT. The results of the treatment showed a reduction in GOR by 70%, and it was reported that the payout was less than a month. However, a reduction in oil production rate was observed. The authors attributed such a reduction to the screen blockage caused by the gel system combined with filter cake developed by the temporary plugging agent. The PAtBA/PEI system was also applied in the North Sea to remediate a leak located between the production tubing and the annulus [49]. The treatment was conducted into a cased vertical wellbore, 93 °C BHT, 1900 psi BHP, and a depth of 5980 ft. The same temporary plugging agent implemented in the previous study was applied ahead and behind the gel treatment. In this treatment, 28 bbl of the polymer gel was injected down the annulus and displaced with 106 bbl of base oil to the desired depth. The treatment results showed that the annulus pressure dropped from 1305 to 350 psi. The gel system was also deployed for treating a naturally fractured carbonate reservoir in Cantarell field, Southern Mexico [50]. The objective of the treatment was to seal a perforated interval (131 ft) located near the gas–oil contact (GOC) and to perforate the lower zone to enhance the oil recovery. The matrix permeability of the formation was around 1–10 md at BHT = 93 °C, and a total of 692 bbl of the gel system was injected. The overdisplacement of the gel system inside the fracture was prevented through multiple injections of gel systems containing silica flour and foam cement. The well underwent acid stimulation after the treatment. The outcomes of the treatment showed a reduction in GOR by 79%, and the oil production rate was 3900 BOPD for 6 months.

##### Other Organically Crosslinked Polymer Gels 

Table 7 summarizes the outcomes of other organically crosslinked polymer gel systems applied for water and gas shutoff treatments.

Dovan and Hutchins [34] applied hydroxypropyl guar (HPG) crosslinked with titanium chelate [53] in treating excess water in Northern California gas wells. Two techniques were adopted in the field deployments to selectively reduce the water permeability and maintain the gas productivity. Those techniques involved the generation of gas channels (through releasing CO_2_) within the emplaced gel by adding potassium bicarbonate to the polymer solution and exposing it to acid. The second technique involved the addition of ester to the gelant containing bicarbonate. The ester is hydrolyzed with temperature increase to generate acid which reacts with the bicarbonate to generate CO_2_ gas channels in situ. The deployment of the first technique was unsuccessful because both gas and water production rates were reduced. For the second technique, there was a short-term reduction in water rate, but the water production rate increased significantly after six months. 

Hutchins et al. [51] reported field applications of a gel system consisting of HPAM crosslinked with a combination of hydroquinone (HQ) and hexamethylenetetramine (HMTA)) organic crosslinkers. The developed gel is stable for 12 months at 149 °C and 5 months at 176.7 °C. One treatment was performed in New Mexico. The reservoir temperature was 121 °C at a depth of 17,000 ft. The objective of the treatment was to seal a cement crack causing a high water influx. In this treatment, 620 bbl of gel was placed inside the production tubing, and the final gel stage was displaced from the wellbore with N_2_. The first attempt of putting the well into production failed; therefore, CT was required to revive the well. The treatment results showed a reduction in water production rate by 60%, but no gas production improvement was observed for 8 months. Hutchins et al. [51] also reported that three wells in Canada were treated with the gel formulation. The reservoir temperature was 113 °C. The results of the combined wells after the treatment showed a reduction in water rate by 65% and an improvement in the gas rate by 315%.

Llamedo et al. [52] reported the results of the deployment of Multigel, developed by PDVSA-Intevep, in treating production well with an excess gas production located in the northeast of Venezuela at BHT = 148 °C. A combined gel–cement squeeze was used to block two perforated gas layers located at the top of the oil-producing interval. Each layer was treated separately due to the reservoir heterogeneity. The treatment results indicated a reduction in GOR by 70% and an increase in the oil rate by 22%.

### 2.3. Inorganic Gel Systems

#### 2.3.1. Lab Evaluation

Karadkar et al. [54] evaluated a nanosilica-based fluid system for matrix gas shutoff treatment (Table 8). The system, which consisted of a mixture of colloidal silica and activators, formed in situ glass-like material upon the interaction with N_2_ inside the porous media. The plugging efficiency of the system in response to both brine and nitrogen gas was examined using a 370 md core. The results indicated that the system exhibited a complete pore plugging for brine at 1500 psi and N_2_ at 600 psi.

#### 2.3.2. Field Applications

Both sodium silicate and a delayed gelation system (DGS) were applied as WSOGs and GSOOs, as shown in Table 9. The sodium silicate was formed through the gelation of a sodium silicate solution and an activator [55]. Herring et al. [56] presented a gas shutoff treatment using activated sodium silicate in the Prudhoe Bay Field, AK. The treatment was deployed to a vertical wellbore with close proximity between the gas and the oil perforated zones. The mechanism of the excess gas was due to tonguing and/or coning through high-permeability sands with permeability ranging from 100 to 400 md. The oil-producing zone was isolated from the injected treatment by a temporary bridging agent. Then, the system was injected through CT into the target perforation zone. The results of the treatments indicated the system was not efficient in blocking the gas perforated zone, and multiple squeezes were needed. However, the sodium silicate system is generally sensitive to fluid compositions, and the presence of contaminations could impact the setting time of the gel system. Chenevière et al. [57] reported a WSOG using a delayed gelation system (DGS) combined with a microcement squeeze. The DGS system was selected based on its high injectivity in a low-permeability formation (1–100 md). A combination of drillable bridge plug/retainer was implemented to isolate the gas-producing zone. The treatment volume consisted of 139 bbl gel +1.9 bbl microcement injection with CT. The water production rate was reduced by 65%, but the gas production declined to 35% after 3 months. The authors attributed the loss of gas productivity to the post injection milling operation during the wellbore intervention.

## 3. Polymer Systems

Water-soluble polymers have been studied and applied to control excess water in gas production wells. These systems perform relative permeability modification (RPM), which is also referred to as disproportionate permeability reduction (DPR). This phenomenon ideally aims to selectively reduce the water permeability, while causing a minimal effect on the oil/gas permeability. The polymer adsorption on the rock pore walls is considered to be the underlying reason for this phenomenon [58,59,60]. Besides polymer adsorption, several mechanisms that contribute to polymer selectivity, including swelling/shrinking [61] fluid partitioning [62], lubrication, and steric and wettability effects [63], have been discussed and debated among scholars. 

The polymers which are applied in the shutoff treatments are classified as synthetic polymers and biopolymers [64]. The selection is based on the reservoir rock properties, temperature, and salinity [16]. Hydrolyzed polyacrylamide is the common synthetic polymer system that has been widely studied and applied [64]. However, the system was reported to be unstable at 75 °C, especially in reservoir formations containing divalent cations such as Mg^2+^ and Ca^2+^ [65,66]. Thus, other polyacrylamide-based polymer derivatives systems have been applied to harsh-condition reservoirs [67]. These polymers are based on the copolymerization and terpolymerization of acrylamide with other anionic monomers (functional groups), such as acrylamide/2-acrylamido-2-methylpropane sulfonic acid (AM/AMPS) copolymer and VA/AM/AMPS terpolymer. The polymers containing a sulfonated monomer can improve salt resistance of polymer systems, especially for formation water containing divalent cations. Biopolymers can be applied in reservoirs with higher salinity and moderate temperature [16]. Xanthan gum and scleroglucan are common biopolymer systems that were reported to be thermally stable at 80 °C (170,000 ppm) and 100 °C (30,000 ppm), respectively [65]. 

Several polymer systems were screened and evaluated at the laboratory scale. Generally, the performance of these systems is evaluated based on the target formation permeability, salinity, and temperature [68].The parameters that determine the feasibility of a certain polymer system include the degree of polymer adsorption, injectivity (resistance factor F_r_), and polymer selectivity. In addition, these parameters are significantly influenced by the polymer’s physical properties such as molecular weight (MW), polymer concentration (P_con_), degree of hydrolysis, and the polymer system electric charge (nonionic, anionic, and cationic) [69].Table 10 summarizes the experimental parameters, conditions, and outcomes for the polymer systems which were evaluated for controlling excess water in gas production wells (WSOGs).

### 3.1. Polymer Systems for Low- to Medium-Temperature Reservoirs—Lab Evaluation

#### 3.1.1. Nonionic and Anionic Polymer Systems

Zaitoun and Kohler [58] claimed that polymer adsorption on the rock pore walls is irreversible, and the efficiency of the adsorption is related to the thickness of the adsorbed polymer layer formed. Zaitoun et al. [59,77] developed two polyacrylamide-based processes (Process A and Process B) that initiate in situ swelling of the adsorbed polymer layer on the pore walls. Process A involves the use of an anionic HPAM with a salinity gradient between the injected and the reservoir formation water, while Process B includes the use of a nonionic PAM injected with a swelling agent to induce hydrolysis in situ. Both processes were evaluated for controlling excess water for low-temperature (36 °C) gas storage wells. The selectivity of Process A was examined using HPAM with a polymer concentration of 2500 ppm injected into a sandstone core with a permeability of 150 md. The selectivity of Process B was evaluated using a 108 md limestone [70]. The results of both studies, demonstrated by gas/water relative permeability curves, showed a significant reduction in water relative permeability K_rw_ after the in situ swelling of polymers with a minimum effect on the gas relative permeability K_rg_. In addition, in both processes, an increase in the residual water saturation S_wi_ was observed after the polymer treatment. Elmkies et al. [78] studied the effect of nonionic PAM adsorption on the selectivity of gas/water systems. Two types of sandstone cores, Vosges (47–63 md) and Beara sandstone (318–392 md), were used in the study. After the injection of 2500 ppm PAM, the reduction in permeability to gas (N_2_) was examined in two conditions: cleaned (no free polymer) and full core (free polymer + adsorbed polymer). The outcomes of the study showed that the RPM mechanisms in the case of the gas/water system are higher compared to those in the oil/water systems experiment conducted under the same experimental conditions. In addition, the F_rrw_ was between 21 and 67 for the treated core, and the Frrg was close to 1. Additionally, the results exhibited that the F_rrg_ was slightly higher for the full core as compared with the cleaned core. However, the measurements were conducted at different permeabilities which could affect the results, and the temperature at which the measurements were conducted was not given.

The feasibility of applying HPAM to selectively reduce excess water in low-permeability reservoirs was also studied. Tielong et al. [71] examined the performance of anionic HPAM using cores obtained from the Lunyu reservoir, with a permeability range of 0.0123 to 0.0224 md. The study evaluated the effects of MW, polymer concentration, and permeability on both polymer adsorption and selectivity. The results showed that for the cores treated with a higher MW (HPAM-2), at the same polymer concentration and core permeability, higher adsorption values and better selectivity were obtained. In addition, no significant increase in the retention values and selectivity were observed with respect to the increase in the polymer concentration between 800 and 1200 ppm. In addition, the S_wi_ increased after the polymer treatment. The authors attributed this increase to the pathway segregation and wall effect. However, the polymer resistance factor values were not presented in the study. It would be valuable to examine whether the polymer system underwent any mechanical degradation during the extrusion from the low-permeability cores. 

#### 3.1.2. Cationic Polymer Systems

In addition to anionic- and nonionic-based polymers, cationic-based polymers were also reported for mitigating excess water in gas production wells. Cationic polymers can strongly interact with the negatively charged rock matrix, which leads to high polymer adsorption [61,79,80]. Dovan and Hutchins [34] investigated whether the adsorption formed by a cationic polyacrylamide without a crosslinker could selectivity reduce the water permeability. After the polymer treatment, the water permeability was decreased by 50% with no effect on the gas permeability (F_rrw_/F_rrg_ = 2.1). However, after the injection of 20 pore volumes, the water permeability was increased (F_rrw_/F_rrg_ = 1.05), indicating that the polymer was flushed from the core. Chiappa et al. [72] screened different types of polymer systems for treating a gas well located in Italy at 48 °C reservoir temperature. The screening was based on the affinity of the polymer adsorption on reservoir sand, which was mainly composed of silicates and alumino-silicates. Among those systems, two cationic polymers (CAT1 and CAT2) and one nonionic polymer were further evaluated owing to their high adsorption values. The cationic polymers showed a higher affinity on the sands taken from the field (2152 to 3694 µg/g). In addition, the selectivity test measured at the end-point permeabilities to brine and gas indicated that a better performance can be achieved with CAT1. The study also showed that there were two cases where the S_wi_ increased after the polymer treatment. Such an increase was associated with a reduction in gas permeability. Burrafato et al. [37] reported further evaluation of the cationic polymer system CAT1. The results from the flow experiments, conducted on a sandpack model with an absolute permeability ranging from 15 to 474 md, showed that the selectivity of the polymer was slightly related to the absolute permeability. In addition, the study showed that the selectivity was hindered when the polymer was shear-degraded prior to injection, especially for the core with permeability below 300 md. Despite the high adsorption values reported in the study, no clear enhancement was observed in terms of the polymer selectivity. Cationic copolymers were also studied to treat medium- to high-salinity gas storage wells [73]. The outcomes of the study showed that the copolymer with a concentration of 1000 ppm resulted in higher adsorption (941–1637 µg/g) on the cores obtained from the reservoir. Additionally, the selectivity of the polymer was a function of both the core permeability and temperature. However, the dimensions of the cores applied in the study were not provided. Al-Shajalee et al. [17] examined the effect of polymer concentration (1000–8000 ppm) and rock permeability (350–462 md) on the selectivity of a cationic poly(acrylamide-co-diallyldimethylammonium chloride) (P(AAm-co-DADMAC)) polymer system. The results of the study, which was conducted using sandstone cores, indicated that the selectivity of the polymer system decreased with the increase in the polymer concentration and rock permeability. Another study was carried out using the cationic P(AAm-co-DADMAC) in order to determine the permeability range at which the RPM treatment is effective [74]. Sandstone cores with low (2.7–66.4 md), medium (350–385 md), and high (3001–5035 md) permeability ranges were deployed in the study. The outcomes of the study showed the highest selectivity was obtained in the medium permeability range. However, in both studies, both polymer adsorption and injectivity were not provided. In particular, the polymer system applied exhibited a shear thickening behavior.

### 3.2. Polymer Systems for High-Temperature Reservoirs—Lab Evaluation

A few applications of polymer systems for harsh reservoir conditions were reported. Ranjbar et al. [76] evaluated the compatibility and selectivity performance of three polymer systems for treating low-permeability, high-salinity, and high-temperature (90–130 °C) wells. These systems include polysaccharide, anionic AM/AMPS copolymer, and two terpolymer systems (Hostadrill and Hostamer, manufactured by HOECHST AG, Germany). The results of the study showed that both the polysaccharide and the anionic copolymer had relatively low adsorption values (10–50 µg/g) and high resistance factor values, which could cause injectivity issues for the low-permeability reservoir. The terpolymer systems, on the other hand, showed higher adsorption values (148–203 µg/g) and better injectivity performance. The selectivity of both terpolymer systems was found to be related to the permeability range, reservoir temperature, and molecular weight. However, the dimensions of the cores were not provided in the study. Okasha et al. [44] evaluated the performance of both a vinyl amide/vinyl sulfonate terpolymer (Polymer A) and a biopolymer (Polymer B). The study showed that the terpolymer has better injectivity compared with the biopolymer, but the selectivity of the biopolymer was higher with F_rrw_/F_rrg_ 11.6–10.15, as compared with 1.14 to 10.1 for the terpolymer system. In addition, the study showed the performance of the gelling system was superior to the polymer systems. However, the comparison was based on different permeability and polymer concentration ranges.

### 3.3. Polymer System Applications for Water Shutoff Treatments in Gas Production Wells

Table 11 highlights the types of polymer systems, reservoir parameters, and treatment results. 

The polymer systems are usually bullheaded to the target formation, thus lowering the cost associated with the mechanical zone isolation. Additionally, the polymer systems can be applied in wells where the polymer gel systems are not appliable, such as gravel-pack completion and for treating microlayered formations [61,83]. Additionally, the polymer systems are preferable when treating low-permeability formations because they exhibit good injectivity which enables them to penetrate deeper into the formation. While the polymer gel systems can be applied for treating matrix- and fracture-related conformance problems, the polymer systems are mainly applied to treat matrix-related conformance problems. However, their plugging performance is less efficient when compared to the polymer gel systems. Many publications have provided guidelines according to which the RPM can be best applied [77,83,84,85]. The polymer systems can be applied to treat wells with excess water production originating from high-permeability streak and water coning problems [81].The polymer injection is overdisplaced with N_2_ to push the nonadsorbed polymer into the formation and to restore the gas productivity [37].

Most of the RPM field applications reported in the literature were applied for gas storage wells. The mechanisms of the excess water in those wells usually originate from a high-permeability streak and water coning problems. Zaitoun et al. [59] reported that Process A was implemented to treat a 197 ft sandstone formation with a permeability contrast ranging from 10 to 3000 md. A total of 4400 bbl of HPAM at a concentration of 3000 ppm were bullheaded to the target zone. The treatment resulted in a reduction in the WGR, while no changes were observed in terms of the gas injection and production. Process B, PAM + KOH, was utilized to treat a 92 ft multilayered, high-salinity formation with permeability contrast (10–700 md). Due to the incompatibility between the swelling agent and the bactericide injected, a premature crosslinking of the polymer at the surface and in the wellbore occurred, leading to formation damage. Three wells were treated with the anionic HPAM-2 in a moderate-temperature (75 °C), low-permeability reservoir. The reservoir consists of three bearing zones with an average permeability range from 7 to 124 md [71]. A total of 4088 bbl was injected into the low-productivity zones. The results of the treatment showed a reduction in the water cut by 60 to 70% and an increase in the gas production by 10 to 20% for 1.5 years.

Burrafato et al. [37] reported the use of cationic polyacrylamide in treating shaly, low-permeability sands in a low-temperature multilayered reservoir. The treatment consisted of the injection of 128 bbl of CAT1 through CT to reduce the injectivity during the wellbore cleaning, and then a total of 220 bbl of CAT1 was bullheaded to the target layer (14.8 ft). After treatment, the water production rate kept increasing. The analysis of the back-produced water using total organic carbon (TOC) showed that 325 ppm of polymer concentration was initially produced, and then the polymer stabilized at a concentration of 50 ppm throughout the reported production response for the treated well. Two gas storage wells were treated with the cationic copolymer [73]. The wells exhibited high permeability contrast ranging from 40 to 3600 md, and the average thickness of the wells was 69 and 112 ft, respectively. Around 1300 bbl were injected into treated zones. The results of those treatments indicated an improvement in gas productivity. However, due to the sessional injection and production of those gas storage wells, the actual performance of the DPR treatments cannot be easily identified. It is unclear whether the thickness of the treated zones contained any crossflow or not.

The Hostamer and Hostadrill terpolymer systems were applied to control excess water in high-temperature reservoirs (90–130 °C) with a formation permeability range from 10 to 90 md. The Hostadrill terpolymer was implemented in a gas storage well (90 °C) to treat a 43 ft formation that consisted of sandstone with clay intercalations [73,76]. The results of the treatment indicated an improvement in the GWR. A deep reservoir with a high temperature (130 °C) was treated with Hostamer [73]. The formation of the well contained a sequence of both sandstones and shales. After the cementation of the well, the re-perforated layer with a 17 ft thickness, producing water and gas, was treated with 1258 bbl. In addition, a gas-producing layer located beneath the treated layer was re-perforated (7 ft). The results of the treatment showed an increase in the gas production rate. However, the performance of the RPM cannot be solely identified due to the contribution of the perforated gas-producing layer to the treatment results. The terpolymer system was also applied to treat a 10 ft interval, low-permeability gas-producing well in Canada [81]. A polymer concentration of 30,000 ppm was implemented. The treatment resulted in a loss of gas productivity. The well was re-opened for production after two months; the gas production resumed, resulting in a reduction in the WGR for a 5-month period.

Polymer systems were also reported to have a synergetic effect of controlling excess water and sand production in gas production wells with unconsolidated formations [86]. Dupuis et al. [82] reported that nine offshore gas-producing wells (40–55 °C) were treated with a synthetic copolymer. The injected volume ranged from 251 to 403 bbl for 10 to 50 md formations. Three of the treated wells showed a reduction in both sand and water production and a gas productivity improvement. Zaitoun et al. [75] presented another field treatment conducted using a synthetic copolymer in a gravel-packed gas-producing well. Based on the laboratory evaluation, the synthetic copolymer exhibited an adsorption of 600 µg/g and a selectivity (F_rrw_/F_rrg_) of 5.90. A total of 283 bbl with a 1500 to 3000 ppm ramp-up polymer concentration was injected to treat 43 ft formation thickness. The treatment succeeded in extending the gas well productivity, but no improvements were observed in terms of water reduction. 

## 4. Current Challenges and Recommendations for Future Work

The chemical conformance control treatments related to natural gas are challenging because of the nature of the reservoir conditions accompanied by the natural gas properties. While a number of review papers related to the polymer and polymer gel systems have been published, none of them have focused on the compatibility and efficiency of these systems applied for natural gas-related conformance control. Our summaries of the lab evaluations and field applications indicate that a limited number of polymers and polymer gels have been investigated and applied in the field. Various experimental methods have been used to evaluate the properties of the polymers and polymer gels to determine whether they can be used for conformance control treatments. There exist some limitations in the evaluation methods which could affect the practical performance of these systems in real reservoir conditions. This section presents the limitations of the current polymer gel and polymer systems and testing methodology, from which future prospects are provided in terms of product development and evaluation.

### 4.1. Limitations of the Existing Systems

**Polymer Gel Systems:** Among the polymer gel systems reported in public literature, HPAM/Cr(III) and PAtBA/PEI were the most evaluated and applied as WSOGs and GSOOs. The HPAM/Cr(III) system was claimed to be H_2_S-resistant; however, there are no systematic evaluations being carried out to investigate such a claim. Additionally, HPAM/Cr(III) is subjected to hydrolysis at elevated reservoir temperatures, especially in the presence of divalent cations such as Mg^2+^ and Ca^2+^ [87]. Albonico and Lockhart [88] reported that the working temperature of this system is safely limited to up to 82 °C. Another drawback of the HPAM/Cr(III) system is related to the ineffective in-depth transportation of the Cr(III) due to its significant retention, specifically in carbonate reservoirs [89]. The PAtBA/PEI system was the most studied and applied organically crosslinked polymer system. The system was reported to be resistant to acid and stable in H_2_S and CO_2_ environments, but there are no studies within the literature to support this claim either. Furthermore, some organically crosslinked polymer gel systems such as HPAM/HQ+HMTA and Multigel were applied in the field without a reported lab assessment of their performance [51,52].The summary of the polymer gel field application indicates that the majority of the current systems have a limited effectiveness period. Additionally, some polymer gel treatments resulted in damage to the hydrocarbon zones, in which acid stimulation was performed to revive the well productivity. Furthermore, the current polymer gel systems demand complicated well intervention for the systems to be placed in the deep, high-temperature, and low-permeability reservoirs, including complex mixing equipment at the surface, precooling of the wellbore to prevent the premature gelation, and tailing in the injected systems either with polymers at high concentration or through some additives such as microcement and silica flour to improve the overall treatment resistance to the near-wellbore conditions.

**Polymer Systems:** Polyacrylamide is a common synthetic polymer system that has been widely studied and applied [64]. A recent study reported that more than 85% reduction in viscosity was observed when polyacrylamide was aged at 130 °C for three months [90]. This implies that any polyacrylamide-based system applied for high-temperature applications is suspectable to thermal degradation with time. The polymer systems were mostly applied to treat gas storage wells. However, these treated wells were continuously exposed to different cycles of gas injection and production [59].Therefore, a conclusion about the effectiveness of these polymer treatments cannot be clearly drawn. Additionally, other treated wells exhibited a reduction in the water production but only for a short time interval, which could be mainly due to the dilution and the desorption of the adsorbed polymer in porous media with the produced water [37]. 

### 4.2. Limitations and Recommendation of Evaluation Methodologies

**The Use of N_2_ to Mimic Natural Gas Behavior:** The performance of polymers and polymer gels has mainly been evaluated using N_2_ instead of natural gas. However, the two gases have different properties, and therefore their interactions with polymers or polymer gels could be different. In addition, many natural gases contain impurities such as CO_2_ and H_2_S. These impurities could adversely impact the performance of these systems.

**DPR/RPM Evaluation:** DPR/RPM is the major feature pursued in many conformance control treatments. However, the DPM/RPM performance is affected by many parameters, mainly including injection mode and sequence, injection rate, rock permeability, remaining oil saturation, polymer properties, and gel compositions. Therefore, it is necessary to provide a comprehensive study when the DPR/RPM property is evaluated. In addition, RPM mechanisms remain unclear and are worthy of further investigation, which will significantly facilitate the design of new polymers and polymer gels.

**Lack of Reports of Key Parameters in Publications:** Gels and polymers are mostly applied for near-wellbore conditions where high pressure, high-pressure gradients, and high flow rates exist. Current studies mainly evaluated their performance at relatively low pressure and flow rate conditions. Therefore, it is necessary to evaluate their performance at near-wellbore conditions to reflect the shear and fluid velocity effects on polymer and gel performance. 

### 4.3. Recommended Future Research Directions

Natural gas-related conformance problems impose significant challenges for many companies during natural gas reservoir development and during natural gas injection. The latter has become a proven technology to improve oil recovery for both conventional and unconventional reservoirs. Therefore, it is of major importance to develop applicable and cost-effective technologies to control the conformance and subsequently improve hydrocarbon recovery. The following breakthroughs and improvements could be significantly important for successful applications of conformance control treatments:

- Development of novel polymers and polymer gels that can be used in harsh conditions, including high temperature, high salinity, CO_2_ and H_2_S environments, large fractures, or fracture-like features such as wormholes.
- Selective shutoff treatments: Current treatments mainly consider the role of polymers and polymer gels on gas, oil, and water permeabilities modification; however, the selective penetration of a polymer or a gelant system during its injection is vital to prevent damaging the unswept zones and consequently improve the treatment efficiency.
- Systematic investigations on the feasibility of applying gels in natural gas-related conformance problems. Gels have been widely investigated and applied to control water production in oil reservoirs. However, there is a limited number of published studies regarding both water control from natural gas reservoirs and natural gas production control from oil reservoirs. A series of studies should be conducted, including the effect of the natural gas and its impurities (CO_2_ and H_2_S) on the gelation kinetics, gel strength and thermostability of the polymer gel systems, DPR/RPM performance when applied for selective water or gas shutoff, and adsorption/chromatographic effect when used for in-depth treatment.
- Development of numerical simulation tools to optimize gel treatment design. Currently, all commercial software packages are incapable of properly describing the gel or gelant injection performance during transport through common porous media and fractures and are unable to quantify the gel plugging performance, which is affected by many parameters.

## 5. Summary

Shutoff treatments in natural gas-related production wells are challenging due to the harsh reservoir conditions and the properties of the natural gas. In this paper, a comprehensive review of the laboratory evaluation and field applications of using polymer gel and polymer systems as WSOGs and GSOOs is presented. The summary of the polymer gel lab evaluation shows that HPAM(III) and PAtBA/PEI were the most evaluated and applied polymer gel systems for WSOGs and GSOOs. The results of the lab evaluation also show that some polymer gel systems exhibit a certain degree of selectivity, but the selectivity progressively decreases due to the polymer gel breakdown. The evaluation of the PAtBA/PEI system indicates that the system has relatively low water and gas breakthrough pressure. The lab evaluation for the polymer systems focuses on their selectivity. Several polymer systems such as nonionic and cationic PAM, HPAM, copolymers, and terpolymers are discussed. However, the performance of polymer gel and polymer systems are studied using N_2_ to replicate the behavior of natural gas. In addition, other parameters including the long-term gas exposure effect on the stability of the systems and the effect of the extended gas and brine flooding are not evaluated for those systems. Different techniques were applied during the polymer gel treatments such as alternate injections of gas (N_2_) and gelant for WSOGs and a combination of cement and gel squeeze for GSOOs. However, the outcomes of the treated wells were reported only for a short time interval. The polymer systems were mostly applied to treat gas storage wells, and terpolymer systems were used as RPM for reservoirs with a temperature ranging from 90 to 130 °C.

## Figures and Tables

**Table 1:** Inorganically crosslinked polymer gels—lab evaluation.

Ref.	Polymer Gel	Model Parameters			Operating Conditions			Outcomes
		Lithology	D in.	L in.	Gas	p psi	T °C	
[34]	4000 ppm Anionic PAM (dichromate/thiosulfate) Cationic PAM (chromium or aldehydes) Lignosulfonates/chromium Silicates/acidic salts TDS: seawater	Sandstone K (383–541 md) Φ (23%)	1.0	5.0	N_2_	290	59	Anionic PAM gel F_rrw_ (17.4): F_rrg_ (2.6) F_rrw_/F_rrg_ (6.7) Cationic PAM gel F_rrw_ (13.4): F_rrg_ (2.0) F_rrw_/F_rrg_ (6.7)
[35]	13,900 ppm HPAM/212 ppm Cr(llI) (66/1) TDS: 10,000 ppm	Sandstone K (650 md) Φ (21%)	1.4	5.5	N_2_	1500	41	F_rrw_ (170,000): F_rrg_ (284) F_rrw_/F_rrg_ (599) ↑WAG_cycles_ ↓F_rrw_/F_rrg_ (22)
[36]	5000 ppm HPAM/800 ppm Cr(llI) (7/1)	Artificially fractured carbonate K (54.3–156 md)	1.5	-	CH_4_	-	-	F_rrw_/F_rrg_ (8.75) ↑WAG_cycles_ ↓F_rrw_/F_rrg_ (7.75)
[37]	35,000 ppm HPAM/900 ppm Cr(llI) (40/1)	Sandstone K (170 md)	-	-	-	-	37	↓ K_w_ (70%) ↑ K_g_
[19]	3000–6000 ppm HPAM/Cr(III) (40/1)	Crushed carbonate core K (10.000–20,000 md)	-	-	-	-	-	F_rrw_/F_rrg_ (1.5 to 2)
[38]	HPAM/Cr(III) (40/1)	Berea cores K (400–600 md) Fractured cores Kf (6000 md) Φ (24%)	1.5	4	N_2_	507	RT	F_rr_ f(remaining gelant in the core and flooding history)
		Sandpack Crushed limestone K (24 d) Φ (36%)	1.5	11.4	N_2_	507		
[39]	20,000 ppm P(AAM-co-AA)Na/200–600 ppm Cr(llI) (100/1) TDS: 20,000 ppm	Sandstone K (140–170 md) Φ (18.7–20%)	1.5	3	N_2_	500–1500	60	↑ Cr(llI)_con_ ↑ F_rr_ ↑ Q_w_ ↓ F_rrw_ (shear thinning) ↑ Q_g_ ↑F_rrg_ (shear thickening) ↓ F_rrw_/F_rrg_ ↑ Cr(llI)_con_ ↓ Q
	20,000 ppm P(AAM-co-AA)Na/300 ppm Cr(llI) (66/1) TDS: 20,000 ppm	Micromodel K (2500 D) Φ (48%)	0.4	0.8	N_2_	500	24	
	20,000 ppm P(AAM-co-AA)Na/600 ppm Cr(llI) (33/1) TDS: 20,000 ppm	Capillary tube	0.02	6	N_2_	-	-	
[40]	90,000 ppm PAM with aluminum (acetate, amino-acetate, nitrate, and lactate)	Plugging efficiency using API PPT with fracture disc of 1 mm width	-	-	-	-	-	PAM/aluminum acetate was selected Gelation time (50 min) at 75 °C It crosslinked with PAM at a wide range of pH (3.5 to 8.5) Sealed the fracture under 700 and 2000 psi

**Table 2:** HPAM/Cr(III)—water shutoff treatments in gas production wells (WSOGs).

Ref.	Field/ Wellbore	Mechanism of Excess Water	Reservoir Parameters					Treatment Design				Results
			Formation	BHP Psi	BHT °C	Depth ft	Salinity ppm	Type	P_Con_ ppm	TDS ppm	Vol bbl	
[34]	North Mexico /vertical	Water influx (fracture-related)	Sandstone	1500	59	-	Seawater	Anionic PAM (dichromate/ thiosulfate)	4000	-	634	Q_w_↓(93.8%) Q_g_ ↓(84%) 3 years
[19]	Canada/ deviated openhole	Water influx (fracture-related)	-	2031	77	-	-	HPAM/ Cr(III) (40/1)	3000 to 8000	-	802	WGR↓(58%) Qg ↑(71%) 6 months
[37]	Italy/vertical with ICGP	Water table close to perforations	Shaly sands 100 md	1465	37	3727	-	HPAM/ Cr(III) (40/1)	35,000	-	314	Successful for two weeks only
[41]	Northern Arkansas/horizontal casedhole	Layer communication (fracture-related)	Shale	3000	79	7000	-	HPAM/ Cr(III)	5500 to 50,000	-	440	Q_w_ ↓ (97%) Q_g_ ↑ (17%) 2 months

**Table 3:** HPAM/Cr(III)—gas shutoff treatments in oil production wells (GSOOs).

Ref.	Field	Mechanism of Excess Gas	Reservoir Parameters					Treatment Design				Results
			Formation	BHP Psi	BHT °C	Depth ft	Salinity ppm	Type	P_Con_ ppm	TDS ppm	Vol bbl	
[42]	Prudhoe Bay, AK	Gas coning + channeling (matrix-related)	Sandstone	3500	104	8800	-	HPAM/ Cr(III) (60/1)	40,000 50,000	-	93–120	GOR ↓ 6 months then increased to pretreatment level
[43]	Prudhoe Bay, AK	Leaking cement-squeezed perforations	Sandstone K (150–300 md)	3400	85–99	8800	-	HPAM/ Cr(III)	50,000 70,000	-	-	85% success rate based on covering the treatment cost

**Table 4:** Organically crosslinked polymer gels—lab evaluation.

Ref.	Polymer Gel	Model Parameters			Operating Conditions			Outcomes
		Lithology	D in.	L in.	Gas	p psi	T °C	
[35]	30,000 ppm resorcinol/30,000 ppm formaldehyde TDS: 5000 ppm KCl, 4200 ppm NaHCO_3_	Sandstone K (650 md) Φ (21%)	1.4	5.5	N_2_	900	41	F_rrw_ (10,400): F_rrg_ (126) F_rrw_/F_rrg_ (83) ↑WAG_cycles_ ↓ F_rrw_/F_rrg_ (7.9)
[44]	1000–2000 ppm PAtBA/PEI TDS: 218,000 ppm	Carbonate K (10–3000 md) Φ (20%)	1.5	2.0	N_2_	1500	90	F_rrw_ (2.75): F_rrg_ (1.25) F_rrw_/F_rrg_ (2.2)
[45]	PAtBA/PEI	Sandpack (100 mesh, 7.8 md)	-	20	N_2_	865	132	P_Stable_ (425 psi): Frrg (6555)
		Carbonate fractured core (fracture width = 0.002 in.)	0.9	3.3	-	-	132	P_Breakthrough_ (196 psi)
[46]	PAtBA/PEI 150 gpt polymer, 10 gpt crosslinker, 814 gpt of field mixing water, 686 Ib/1000 gal of NaCl (retarder) compared with a new retarder	Carbonate K (2.7 md) Φ (18.7%)	-	-	N_2_	900	116	The new retarder Gelation time (90 min) at 150 °C P_Injection_ (32 psi)

**Table 5:** PAtBA/PEI—water shutoff treatments in gas production wells (WSOGs).

Ref.	Field/ Wellbore	Mechanism of Excess Water	Reservoir Parameters					Treatment Design				Results
			Formation	BHP Psi	BHT °C	Depth ft	Salinity ppm	Type	P_Con_ ppm	TDS g/l	Vol bbl	
[10]	Indonesia /deviated vertical monobore	Water source located at the top of perforation in a well that is still producing	Sandstone K (500 md)	2200	150	11,830	-	PAtBA/ PEI	-	-	-	Q_w_↓(97.5%) Q_g_ ↑(83.3%) 2 months
[46]	Middle East/openhole horizontal	Water was entering the openhole at the toe	Carbonate K (2–3 md)	7000	149	13,611	-	PAtBA/ PEI	250 gal/1000 gal	20	155	Q_w_↓(58%) Q_g_ ↑(672%) 8 months
[47]	Gulf of Thailand/vertical monobore	Water production from top perf zones (matrix-related)	-	-	-	-	-	PAtBA/ PEI	-	-	10	Q_w_↓(49%)

**Table 6:** PAtBA/PEI—gas shutoff treatments in oil production wells (GSOOs).

Ref.	Field/ Wellbore	Mechanism of Excess Gas	Reservoir Parameters					Treatment Design				Results
			Formation	BHP Psi	BHT °C	Depth ft	Salinity ppm	Type	P_Con_ ppm	TDS g/l	Vol bbl	
[48]	North Sea/vertical openhole gravel pack	High K channel between casing shoe bottom and top of gravel pack	Sandstone K (17–340 md)	3539	88	-	-	PAtBA/ PEI	-	-	638	GOR ↓ (70%) Qo ↓ 12 months Payout (less than a month)
[49]	North Sea/vertical casedhole	Communication between the tubing and casing	Chalk K (1–340 md)	1900	93	5980	-	PAtBA/ PEI	-	-	20	The annulus pressure ↓ from 1305 to 350 psi with minimal leak
[50]	Southern Mexico/vertical casedhole	Perforated interval close to GOC/high K streak (fracture-related)	Carbonate K (1–10) md	1400	93	8645	-	PAtBA/ PEI	-	-	660	GOR ↓ (79%) Qo = 3900 BOPD 6 months

**Table 7:** Other organically crosslinked polymer gels.

Ref.	Field/Wellbore	Mechanism of Excess Water/Gas	Reservoir Parameters				Treatment Design			Results
			Formation	BHP Psi	BHT °C	Depth ft	Type	P_Con_ ppm	Vol bbl	
[34]	Northern California/vertical	-	-	-	-	-	Hydroxypropyl guar (HPG) crosslinked with chelated titanium	-	-	No improvement in gas and water rates
[51]	New Mexico	Water influx through crack/fracture in cement	Sandstone	-	121	17,000	HPAM/HQ+HMTA	-	-	Q_w_ ↓ 60%) Q_g_ ↔ 8 months
	Canada	Water influx through fracture	Carbonate K (200 md)	-	113	-	HPAM/HQ+HMTA	-	620	3 wells Q_w_ ↓ (65%) Q_g_ ↑ (315%)
[52]	Venezuela/vertical	Gas channeling and coning	K (54–180 md)	-	148	14,080	Multigel (PDVSA-Intevep)	-	-	GOR ↓ (70%) Q_o_ ↑ by (22%)

**Table 8:** Inorganic gel systems—lab evaluation.

Ref.	Polymer Gel	Model Parameters			Operating Conditions			Outcomes
		Lithology	D in.	L in.	Gas	p psi	T °C	
[54]	Activated nanosilica system 78.5 (wt.%) nanosilica, 0.2 (vol%) surfactant, 0.2 (vol%) clay control	Sandstone K (370 md) Φ (24%)	-	-	N_2_	500	93	Complete pore plugging P_Brine_ = 1500 psi P_N2_ = 600 psi

**Table 9:** Inorganic gel systems—field applications.

Ref.	Field/Wellbore	Mechanism of Excess Water/Gas	Reservoir Parameters				Treatment Design			Results
			Formation	BHP Psi	BHT °C	Depth ft	Type	P_Con_ ppm	Vol bbl	
[56]	Prudhoe Bay, AK/ vertical	Gas coning/tonguing through high K sands	Sandstone K (100–4000) md	-	93	15,000	Activated sodium silicate	-	192	Incomplete shutoff
[57]	Indonesia/vertical monobore	Crossflow from watered-out to gas-producing interval	Shaly sands K (1–100) md	2815	118	10,658	Inorganic gel—delayed gelation system	-	139	Q_w_ ↓ (65%) Q_g_ ↓ (33%) 3 months

**Table 10:** Polymer systems—lab evaluation.

Ref.	Polymer Gel	Model Parameters			Operating Conditions			Outcomes
		Lithology	D In.	L In.	Gas	p psi	T °C	
[34]	Cationic PAM	Sandstone K (383–54 md) Φ (23%)	1.0	5.0	N_2_	290	59	Frrw (2.1): Frrg (1) Frrw/Frrg (2.1) After 20 PV Frrw (1.5): Frrg (1.4) Frrw/Frrg (1.05)
[59]	2500 ppm HPAM 8 × 10^6^ MW TDS: 972 → 8243 ppm	Sandstone K (280 md) Φ (24%)	1.6	3.1	N_2_	-	35	Adsorption (192.4) µg/g Before swelling Frrw (1.8): Frrg (0.5) Frrw/Frrg (3.6) After swelling Frrw (28.6): Frrg (0.5) Frrw/Frrg (57.2) Swi ↑
[70]	2500 ppm PAM + 500 ppm Activator TDS: 14,000 ppm	Limestone K (108 md) 23%	1.6	2.3	N_2_	435	36	Adsorption (150)µg/g Before swelling Frrw (13.8): Frrg (1.5) Frrw/Frrg (9.2) After swelling Frrw (52.8): Frrg (1.3) Frrw/Frrg (40.6) Swi ↑
[71]	1200 ppm Anionic HPAM-2 17 × 10^6^ MW TDS:2362.5 ppm	Sandstone Φ (14.5–16.7%) K (0.0123–0.0870 md)	1.0	2–3	N_2_	-	72	Adsorption (174.5–120.4) µg/g K_ab_ ↓: µg/g ↑ Frrw ↑ Frrg ↓ Frrw/Frrg (5.2–5.0) Swi ↑
	800–1200 ppm Anionic HPAM-1 14 × 10^6^ MW TDS:2362.5 ppm	K (0.0212—0.0224 md)	1.0	2–3	N_2_	-	72	Adsorption (132.6–138.9) µg/g P_CON_: µg/g ↑ Frrw ↑ Frrg ↑ Frrw/Frrg (2.7–1.7) Swi ↑
[37]	2000 ppm Cationic PAM CAT1: 4 × 10^6^ MW TDS: 20,000 ppm	Reservoir sands K (15–474 md) Φ (16%)	1.0	2.6	N_2_	-	48	CAT1 K_ab_ ↑ Frrw ↑ Frrg ↓ Sheared CAT1 K_abs_ < 300 md: Frrw/Frrg (5.4) K_abs_ > 300 md Frrw/Frrg (1.4)
[72]	1000–2000 ppm TDS: 20,000 ppm Cationic PAM CAT1: 4 × 10^6^ MW	Sandpack Reservoir sands Φ (16%)	1.0	2.6	N_2_	-	48	Adsorption (2691–3694) µg/g F_rrw_/F_rrg_ (2.9–3.4) S_wi_ ↑
	Cationic PAM CAT2: 0.8 × 10^6^ MW							(2152–3187) µg/g F_rrw_/F_rrg_ (2.6) S_wi_ ↑
	Nonionic PAM PAM: 5 × 10^6^ MW							(1634–2477) µg/g F_rrw_/F_rrg_ (1.8)
[73]	1000–2000 ppm Cationic acrylamide co-polymer 6 × 10^6^ MW TDS: 216,000–53,000 ppm	Reservoir cores K (938–1473 md) Φ (21–26%)	-	-	N_2_	-	36 23	Adsorption (1637–941) µg/g Fr (25.07–15.04) F_rrw_ (11.34–6.92) F_rrg_ (1.14–1.07)
[74]	1000 ppm Cationic poly(acrylamide-co-diallyldimethylammonium chloride) 25,000 MW TDS: 20,000	Sandstone K (2.7–66.4 md) Φ (17.6–19.5%)	1.5	3.0	N_2_	-	-	F_rrw_ (1.44–2.35) F_rrg_ (4.60–7.60) F_rrw_/F_rrg_ (0.19–0.43)
		K (350–385 md) Φ (21%)						F_rrw_ (2.3–2.86) F_rrg_ (0.90–0.928) F_rrw_/F_rrg_ (2.5–3.08)
		K (3001–5053 md) Φ (23–29%)						Frrw (1.21–1.75) Frrg (1.0–1.32) Frrw/Frrg (1.03–1.75)
[17]	1000–8000 ppm Cationic poly(acrylamide-co-diallyldimethylammonium chloride) 25,000 MW TDS: 20,000	Sandstone K (350–426 md) Φ (21%)	1.5	2	N_2_	1000	RT	F_rrw_ (1.15–2.75) F_rrg_ (0.6–2) F_rrw_/F_rrg_ (1.38–3.96) F_rrw_/F_rrg_ ↑Pcon ↓ K_abs_ ↓
[75]	500–5000 ppm Copolymer (POWELGEL P321) TDS: 33,000 ppm	Sandstone K (360 md) md Φ (29%)	1.5	2.3	N_2_	-	40	Adsorption (600) µg/g F_r_ (2.4–76.6) F_rrw_ (1.5–6.5) @ 5000 ppm F_rrw_/F_rrg_ (6.5/1.1) 5.90 S_wi_ ↑
[76]	1000 ppm Polysaccharide 14 × 10^6^ MW TDS: 60,000 ppm	Vosges sandstone K (40–60 md) Obernkirchr sandstone K (4–9 md) Reservoir core K (10–20 md)	-	-	N_2_	-	90 130	Vosges: 90 °C Adsorption (50) µg/g Fr (35.8)
	1000 ppm Vinyl sulfonate/acrylamide copolymer (AM/AMPS) HMW TDS: 20,000 ppm							Vosges: 90 °C Adsorption (10) µg/g Fr (25.7)
	1000 ppm Vinyl sulfonate/vinyl amide/acrylamide Terpolymers (VS/VA/AM) Hostadrill: 0.5 × 10^6^ MW Hostamer: 1.0 × 10^6^ MW TDS: 300,000 ppm							Vosges reservoir core: 130 °C Adsorption (135–185) µg/g Fr (7–9.5): (5.6–7.6) F_rrw_/F_rrg_ (3.8–5.8): (2.3–5.3) Adsorption (148–203) µg/g F_r_ (11–16): (8.8–12.8) F_rrw_/F_rrg_ (4.6–6.8): (3.0–6.1)
[44]	1000 to 2000 ppm Biopolymer(Polymer B) HMW TDS: 217,000 ppm	Carbonate K (12–138.7 md) Φ (20)%	1.5	2.0	N_2_	1500	90	Adsorption (21–94) µg/g ↑ P_CON_↑ F_r_ (25–44) F_rrw_ (18.5–20.3) F_rrg_ (1.6–2) F_rrw_/F_rrg_ (11.6–10.15)
	1000 to 2000 ppm Vinyl amide/vinyl sulfonate Terpolymer (Polymer A) TDS: 217,000 ppm							Adsorption (20.3–75.4) µg/g Fr (2.4–1.9) F_rrw_ (1.6–1.9) F_rrg_ (1.4–1.4) F_rrw_/F_rrg_ (1.14–10.1)

**Table 11:** Polymer systems—water shutoff treatments in gas production wells (WSOGs).

Ref.	Location	Mechanism of Excess Water	Reservoir Parameters					Treatment Design				Results
			Formation	BHP Psi	BHT °C	Depth ft	Salinity ppm	Type	P_Con_ ppm	TDS ppm	Vol bbl	
[59]	France/gas storage	Water encroachment through active water aquifer to high-permeability streak	Sandstone K (100–5000 md)	-	30	1640	972	HPAM	3000	8209	4400	WGR ↓ and W_Prod_ ↓ G_Prod_ and G_Injt_ unchanged
[77]	France/gas storage	Water coning to multiple layers with good vertical communication	Limestone K (10–700 md)	1450	36	2300	14,015	Nonionic PAM + KOH	2000	River water	1600	No improvement
[71]	China	Water influx through high-K streak	Sandstone K (7–124 md) Φ (11.2%)	-	75	7546	-	Anionic HPAM-2	1000	2363	4088	WGR ↓ (68%) GWR ↑ (230%) 1.5 years
[37]	Italy/ vertical	-	Shaly sands K (44 md)	985	48	3170	-	Cationic polymer CAT1	1500	30,000	345	GWR ↑ (4.1–11.2) Mscf/bbl WGR ↓ (0.24–0.09) bbl/Mscf 8 months
[73]	Germany/gas storage	-	Sandstone (50–2000 md) Φ (17.4–23.2%)	1015	36	x	216,000	Cationic acrylamide copolymer	1000	-	1415	GWR ↑ (269.5–898) Mscf/bbl 5 years
	Germany/gas storage	-	Sandstone K (40–3600 md) Φ (24–27)%	551	23	x	53,000	Cationic acrylamide copolymer	750–2000	-	1258	GWR ↑ (330–1061) Mscf/bbl 6 years
	Germany/gas storage	-	Sandstone K (50–90) md Φ (14.6–21.3%)	3988	90	7195	270,000	Anionic VS/VA/AM terpolymer (Hostadrill)	500–2000	-	917	GWR ↑ (390–1605) Mscf/bbl 6 years
	Germany	Abandoned gas well loaded up with water	Sandstone K (10–15 md) Φ (11.5–14.7%)	2030	130	11,286	300,000	Anionic VS/VA/AM terpolymer (Hostamer)	1000	180,000	1258	GWR ↑ (0–1) Mscf/bbl 4.5 years
[81]	Canada	-	Sandstone K (25 md)	-	-	-	-	Terpolymer	30,000	-	-	WGR ↓ (0.06–0) bbl/Mscf 5 months
[82]	Adriatic Sea	Water encroachment	Sandstone K (10–50 md)	-	40–55	5584–10,653	30,000–40,000	Copolymer	750 to 1000	30,000	251–403	9 wells were treated
[75]	Adriatic Sea	Water encroachment through high-K streak	Sandstone K (660 md) Φ (36%)	2343	40	1924	33,000	Copolymer	1500 to 3000	30,000	283	Improved the gas production decline rate

## Nomenclature

WSOG	water shutoff treatment in natural gas production wells
GSOO	gas shutoff treatment in oil production wells
BHT	bottomhole temperature (°C)
BHP	bottomhole pressure (psi)
TDS	total dissolved solids (ppm)
CT	coiled tubing
Frrw	water residual resistance factor
Frrg	gas residual resistance factor
Fr	resistance factor
ICGP	inside casing gravel pack
P_Con_	polymer concentration (ppm)
Kf	fracture permeability (md)
Mscf/d	thousand standard cubic feet per day
RT	room temperature (°C)
BWPD	barrel of water per day
WGR	water–gas ratio (bbl/Mscf)
GOC	gas–oil contact
Qg	gas production rate
Qw	water production rate
